# Supplementary material for: Potential mitigating role of ivermectin on the spread of Chlamydia trachomatis by Musca sorbens
Source: PLoS Negl Trop Dis. 2023 Oct 26;17(10):e0011662. doi: 10.1371/journal.pntd.0011662 (PMC10602231; doi:10.1371/journal.pntd.0011662)
Supplement: Attachment — Submitted filename: Response to reviewers.docx [file pntd.0011662.s001.docx]

Sightsavers international
35 Perrymount Road
Haywards Heath
West Sussex
RH16 3BW
UK

Dear Susana, Álvaro and Reviewers

We are pleased to submit a revised draft of the manuscript titled *Potential mitigating role of ivermectin on the spread of Chlamydia trachomatis by Musca sorbens* to PLOS NTD for consideration. We appreciate the reviewers’ feedback and recommendations for expanded analysis for our manuscript. While it has taken a considerable amount of effort, we can see and do appreciate that the outcome is much stronger than previously submitted.

As you will see, we have been able to incorporate changes to reflect the majority of suggestions provided by the reviewers. All changes have been highlighted as track-changes within the manuscript.

We will summarise the changes made in response to the feedback from the reviewers (removing some of the summary text to focus on the action points), with our comments following in colour coded context:

- Green used where the feedback has been implemented in full.
- Blue where we have partially implemented suggestions.
- Red where we have not (including if the suggestion is beyond the scope of the study)

As all reviewers rightfully highlighted the lack of Ethiopian authorship, Fikreab Kebede from the Ethiopian MoH NTD programme has reviewed, contributed revisions and agreed to authorship.

Best wishes

Richard

Reviewer #1:

Yes, yes, mostly, sample size was dictated by available data, partially, no (though please see comment about list of authors) – Fikreab has been reviewed the paper and has kindly agreed to be added as an author.

Reviewer #2:

The main limitation of this observational study is that this difference may be due to a number of potential confounding factors.
The most important of these is the possibility that the prevalence of active trachoma was higher at baseline in the EUs which did not receive ivermectin. This information is available to the authors and should be included in this paper. Other confounders not mentioned in this paper include the number of rounds of azithromycin treatment received in each EU, and altitude, which has been previously shown to be inversely correlated with the prevalence of active trachoma in Ethiopia 1. 1. Alemayehu W, et al Active trachoma in children in central Ethiopia: association with altitude. Trans R Soc Trop Med Hyg. 2005;99(11):840-3 – Addressed. Confounders added to analysis.

Reviewer #3:

This is an ecological/observational study in trachoma control pointing out that evaluation units (EUs) which also received ivermectin MDA had a better trachoma control outcome in terms of recrudescence/persistence of TF.

While this is an interesting observation, and question the hypothesis has been generated by post-hoc consideration of the data. It is not possible to say whether the sample size was adequate or assess power as this depends on how the various competing explanations/confounders would be addressed, although this is likely to be all the data that is available that bears on the question.

The primary analysis presented is a (commendably) simple chi-square comparison of whether an EU received IVM versus the outcomes of trachoma recrudescence and persistence - although maps are presented where some potential confounders are overlaid- EUs receiving IVM were different with respect to land cover and population density and are stated to be similar with respect to access to improved water and sanitation, although no data are presented.

The authors have not performed any kind of modelling or analysis but are just asking us to eyeball the maps. I am sure they could do better by attempting a more formal statistical analysis and evaluating the role of these and other potential confounders at EU level or possibly at a finer level of detail, if they have access to cluster level data – Modelling has been conducted and added.

Technically data 'obtained through the ITI' should respect the principle that all data compiled by the ITI belongs to the contributing Ministries of Health. I am not sure but presumably the same is true of data accessed through ESPEN and so for regulatory purposes there should be a statement that the Ethiopian MoH has sanctioned the accessing of its data for this purpose and takes responsibility for it? – the MoH’s NTD lead contribution as author addresses this.

Results

Reviewer #1: Yes, mostly, improvement suggested

Reviewer #2: See comments above.

In figure 3, which compares the landcover, population density, access to improved sanitation and access to improved water between EUs with and without persistent or recrudescent trachoma, and in figure 2, it is not possible to distinguish between these two groups. These figures should be reformatted to make the difference clear. – Both figures have been revised

Reviewer #3: The lack of an analysis plan, or indeed of an analysis specifically designed to evaluate confounding is a weakness as outlined above - addressed

The maps are well presented and the basis for the observations are clear – maps have been refined

It would help to provide EU level data in a supplement so that independent modeling could be done – all source data are openly available. As we are seeking to continue this study, we wish to release all datasets that we have been working from in future publications

Conclusions

Reviewer #1:

Yes, no (suggestions given), yes, yes

Reviewer #2:

The main limitation of this observational study is that this difference may be due to a number of potential confounding factors.

The most important of these is the possibility that the prevalence of active trachoma was higher at baseline in the EUs which did not receive ivermectin. This information is available to the authors and should be included in this paper. Other confounders not mentioned in this paper include the number of rounds of azithromycin treatment received in each EU, and altitude, which has been previously shown to be inversely correlated with the prevalence of active trachoma in Ethiopia 1. - Addressed

The public health relevance of the observation that districts receiving ivermectin in addition to azithromycin is clear, since there are many districts in Ethiopia in which the prevalence of active trachoma remains high in spite of many rounds of azithromycin mass treatment. As stated in the paper, further research is needed to establish whether the concentration of ivermectin in human faeces is sufficient to inhibit the breeding of M. sorbens – Agreed, we intend to swiftly follow this paper with further research

Reviewer #3:

No!. In my view the authors have no clear path to implicating an effect on M.sorbens reproductive capacity in the data. – Strikingly conflicting with reviewer 1 and 2.

Transmission of trachoma by M.sorbens has not so far been demonstrated at scale in Ethiopia (though I am aware of some preliminary data from Oromia). Short term sterilising effects mediated via dung in IVM treated cattle on reproduction of M.nevili (an unrelated species- there are huge differences in behaviour among Muscids) and the observation that there might be fewer maggots in public toilets beg all sorts of questions (cattle receive much more IVM than humans, M sorbens is not considered to breed effectively in latrines and the appearance/disppearance of unspecified maggots utterly non-specific given the diversity of flies documented as emerging from/breeding in public toilets in various settings). – Outstanding questions are good. We are specifically looking to answer them as best we can and close the knowledge gap.

The authors do not explain how the IVM MDAs were done, but it would be conventional to exclude under 5s , a group which contributes to open defecation and probably under-utilises public toilets or household latrines, because of a paucity of safety data. So I find the argument that this mechanism is definitively 'suggested' by the data as unduly speculative - somewhat beyond the scope of this initial investigation, we are aware of the IVM dosing age and that children also practice OD. We re-stress that this was a first look to assess an initial concept

Possible confounders of the observed association need to be properly addressed- although there have rarely been formal studies of transmission there is generally considered to be less trachoma transmission in 'forested' areas than in more arid places and there are probably a wide range of socioeconomic and behavioural factors that differ between them. – Modelling added.

In general the use of EU level data smoothes out this kind of detail

Editorial and Data Presentation Modifications?

Reviewer #1:

Thank you for the opportunity to review this extremely interesting paper. An important overall comment is that although this analysis used publicly-available data, they are data that pertain to Ethiopia alone, it seems wrong that no authors affiliated to Ethiopian institutions – particularly the Ethiopian FMOH, who would have contributed the relevant data to ESPEN – are currently involved. I think that oversight should be rectified. – Addressed.

6: please change to “Trachoma is the most common infectious cause of blindness.” The blindness itself is not infectious. – Addressed.

6: “A disease associated with lack of access to sanitation and low hygiene standards.” This is a sentence fragment: please edit. – Addressed.

8: E stands for “environmental improvement” (not “improvements”) – Addressed.

12, 13, 14, 18 (and elsewhere): onchocerciasis, lymphatic filariasis, ivermectin and albendazole should not be capitalized unless the word concerned starts a sentence. – Addressed.

14: please delete “other”; including it implies that previously mentioned organisms are also insects, which some might interpret as the pathogens responsible for the diseases in the previous sentence– Addressed.

22: “benefit from a higher impact against trachoma” – please change to reflect the actual outcomes studied. – Addressed.

23: please change “among the population” to “MDA” – Addressed.

25: please change “taking the assessment further” to “further work” – Addressed.

28: same comment as in abstract– Addressed.

28: “A disease associated with poverty, which if left untreated leads to damage to the patient’s eye.” This is another sentence fragment. Also: is there only one patient? Is there only one eye? Is the person or people involved a patient or patients before they seek care? – Addressed.

29: I think the authors might mean “culminates” rather than “cumulates” – Addressed.

30: “Trachoma is strongly associated with lack of access to sanitation”. I think this statement should be referenced. There are lots of small studies that demonstrate the association, but I think the largest (and most convincing) are 1. Garn JV, Boisson S, Willis R, et al. Sanitation and water supply coverage thresholds associated with active trachoma: modeling cross-sectional data from 13 countries. PLoS Negl Trop Dis 2018; 12(1): e0006110. and 2. Sullivan KM, Harding-Esch EM, Keil AP, et al. Exploring water, sanitation, and hygiene coverage targets for reaching and sustaining trachoma elimination: G-computation analysis. PLoS Negl Trop Dis 2023; 17(2): e0011103. – Addressed.

31: suggest delete the words “coupled with low hygiene standards” – this could be interpreted as sounding pejorative or judgmental. – Addressed.

33: please change “bacteria” to “bacterium” – Addressed.

43: please change “cease” to “correct” – Addressed.

47: please change “improvements” to “improvement” – Addressed.

55: please delete “Sightsavers” here. The particular funder should have no bearing on interpreting the weight of the research; including it here is likely to be viewed as an advertorial– Addressed.

58: “even” in this sentence sounds a bit breathless. Please edit– Addressed.

60: a prior trial (and cleaner study, in the sense that only the two relevant agents were administered) for coadministration of ivermectin and azithromycin is: 1. Romani L, Marks M, Sokana O, et al. Feasibility and safety of mass drug coadministration with azithromycin and ivermectin for the control of neglected tropical diseases: a single-arm intervention trial. Lancet Glob Health 2018; 6(10): e1132-e8. – Addressed.

63: the abbreviation “TF” has not previously been defined in this manuscript. Persistence and recrudescence of TF have similarly not been previously defined. – Addressed.

65: Ethiopia has not only the highest burden of trachoma, but the greatest recorded incidence to date of persistent and recrudescent TF– Addressed.

68: “ratified” is far too strong here. The definitions are working definitions proposed at a WHO informal consultation. Incidentally, when abbreviated, “WHO” does not take the definite article. – Addressed.

69-70: the phrasing here conflates “trachoma” and “TF”. One is a disease; the other is a specific sign– Addressed.

72: suggest delete the words “the data processing and” – Addressed.

77: the analysis is secondary. The data are not secondary. – Addressed.

89: presumably there were some EUs that did not have persistent TF or recrudescent TF? (As a grammatical aside here, it is not the EUs that are “persistent or recrudescent” – it’s TF in those EUs.) – Grammatical point addressed, point about EUs without persistent or recrudescent is addressed in the processing steps of Fig 1.

91: having defined the abbreviation “EU”, it should be used consistently– Addressed.

101: “To examine the influence of these variables on this investigation available geographic data have been collated for climate (21), landcover (22), population density (23), sanitation and water access (24). These data were overlaid with the plots of EUs within our datasets for examination.” This is important: it's trying to address an important confounder that might explain differences between EUs co-endemic for oncho or LF and those that have only required interventions against trachoma. Details of the way in which this was built into the analysis are necessary; this would ideally be more sophisticated than simply overlaid visualisations. – Addressed with the addition of modelling.

106: “EUs that could not be aligned from ESPEN into GeoConnect were discounted from our dataset prior to assessment commencing”. I may have misunderstood what this actually means, but feel that the number “discounted” should be included in the flow diagram that forms Figure 1. – Addressed in revision.

121: “During the process of merging GeoConnect and ESPEN datasets, 10 GeoConnect IDs from the trachoma dataset matched two ESPEN IDs. To resolve this, one of the ESPEN ID was randomly retained as a match for each GeoConnect ID.” This seems like a rather arbitrary way to resolve the problem. Can a more considered investigative approach be explored? – Resolved, we have revisited and eliminated this issue

124: “Among the 208 EUs retained for trachoma analyses, 28 had received IVM MDA, starting on or after the year of trachoma baseline survey and occurring on or before the last year of survey on trachoma.” The requirement for IVM MDA to commence *after* (or at worst, in the same year as) the baseline trachoma survey seems sensible to appropriately examine differential reductions in prevalence. But this requirement was not apparent in the methods section. It should be.

Tables 1 and 2: Given the amount of data available, use of three significant digits for the percentages provided here is excessive. Please round to whole numbers. – Addressed.

131 onwards: please don’t describe the *EUs* as being “persistent” or “recrudescent”. These labels can and should be applied to the situation of active trachoma in those EUs, but not the EUs themselves. – Addressed.

143: please delete the adjective “continued” here – it’s redundant. – Addressed.

Figure 2 is surprisingly hard to read at a glance, which should be the role of this type of figure. Can the authors please experiment with other ways of displaying the categories to see if they can construct a more digestible visualization? – Addressed, we have revised all figures and displayed data as clearly as possible

149: “Showing rainfall patterns are not vastly different between the EUs receiving IVM MDA compared to trachoma EUs which met the inclusion criteria for this study.” This is a sentence fragment. Please edit. – Addressed.

The analysis of these data and the data shown in Figure 3 is a bit basic; it assumes that any relationship would be visually obvious and ignores possible interactions of the known confounders identified. Suggest further consultation with a statistician. – Addressed with modelling.

170: risk of *active* trachoma – Addressed.

179: “The plasma half-life of IVM in humans being approximately 18 hours following oral administration.” This is a sentence fragment: please edit. – Addressed.

181: “This anticipated active lifespan is validated by the effect on gastrointestinal nematodes ceasing 12 days post dosing in humans.” A reference should probably be provided to support this statement, but even if it is evidenced, I am not sure that it is relevant. The concentrations needed to produce “effect” on nematodes in the lumen or attached to the mucosa of the GI tract are not necessarily the same as those needed to affect viability of flies ovipositing on soil-exposed faeces. - Line removed. Deemed irrelevant and needlessly conflicting on review.

183: “Initial interest focuses on IVM treatment in humans to deliver M. sorbens control, with complimentary laboratory investigation into veterinarian IVM treatment of domestic livestock. Investigation into use in livestock will guide any addition as further support, added in certain field settings if human IVM treatment is not achieving M. sorbens control (26).” This sounds premature, given that it is not yet proven as an intervention, but also misguided, because (as the authors point out in the next sentence), “Evidence from Gambia shows M. sorbens population responsible for trachoma transmission reproduce only in human excreta (5).” Ref 26 is an observational study that is not in any way convincing, and should not be included in a serious discussion of the evidence. The rest of this paragraph is highly speculative and should be deleted or revised. – Revised and sections have been deleted

A paragraph giving serious considerations to the limitations of the study would augment the paper. Some of the limitations are alluded to but should be identified explicitly as limitations, and gathered together in the usual style of scientific papers. – limitations section has been added

Reviewer #2:

A number of points need to be clarified in this manuscript.

TF 1-9 is not defined. – Addressed.

The differences between an impact survey and a surveillance survey, and between a persistent and a recrudescent EU are not clear. – Addressed.

The authors state that trachoma is the leading cause of infectious blindness, but blindness is not infectious. It is the leading infectious cause of blindness. – Addressed.

They state that evidence from Gambia shows the M. sorbens population responsible for trachoma transmission reproduces only in human excreta. This is incorrect. The paper they cite states that “Musca sorbens emerged from human (6/9 trials), calf (3/9), cow (3/9), dog (2/9) and goat (1/9) faeces”. – Addressed.

Reviewer #3:

I am OK with the way they have presented the data but I consider that the analysis and discussion are primary weaknesses – Addressed, univariate and multivariate models added to analysis

The maps are great but not adequate to enable the results to be evaluated independently- could a dataset of all EU level variables examined be appended? – As this is an initial study, full dataset compiled from openly available data will be made available with later publications

--------------------

Summary and General Comments

Reviewer #1:

(No Response)

Reviewer #2:

The fact that evaluation units (EUs) which received mass treatment with ivermectin in addition to azithromycin were less likely to have persistent or recrudescent active trachoma than those which received azithromycin alone is an interesting observation of potential public health relevance.

The main limitation of this observational study is that this difference may be due to a number of potential confounding factors. The most important of these is the possibility that the prevalence of active trachoma was higher at baseline in the EUs which did not receive ivermectin. This information is available to the authors and should be included in this paper. – Addressed.

Other confounders not mentioned in this paper include the number of rounds of azithromycin treatment received in each EU, and altitude, which has been previously shown to be inversely correlated with the prevalence of active trachoma in Ethiopia 1. – Addressed.

Given the fact that the data included in this study were all collected in Ethiopia, it is surprising that none of the authors appears to be from that country. – Addressed.

Reviewer #3:

The observation that on the face of it MDA with IVM may be associated with improved trachoma control outomes at EU level is novel and potentially important. However there are a wide range of potential confounders and missed analytical opportunities and evidence for the speculated mechansism is severely lacking: - – Addressed.

1) is there evidence that M.sorbens transmits trachoma in any of the EUs? – Ascertaining as an absolute certainty this is beyond the scope of the study, but data eludes to it

2) is there evidence that there is less transmission of trachoma underlying the reduced risk of labelling an EU as persistent or recrudescent (tests for infection?) - Beyond the scope of the study

3) is there evidence that any other parameters of fly borne transmission (e.g face-fly contact) or the observations of flies attacking ocular/nasal secretions that may be reduced following IVM MDA? – Beyond scope of this study, will follow in next stages of investigation

4) It would normally be considered that flies are quite successful at reproducing (they breed like flies after all) and there is very little evidence that IVM affects this particular fly to the extent of temporarily altering its behaviour(normally considered to be quite plastic) in ways that would affect its reproductive success for more than a few days – As an initial investigation, this study is the first step in gaining insight on this.

5) Toxicity to egg laying female flies may have more impact than toxicity to maggots (400+ produced each time) – This is a particularly appreciated comment as we had somewhat overlooked it, we have added a line on it into the paper, while maintaining focus on reproduction. Further we are incorporating this into the lab assessment stage. Would be wonderful if both larval development and adult mortality can be influenced by IVM.

6) Is there any association of TF outcome with IVM coverage? – data suggests it, but validation in the field is needed

7) of the very large number of potential confounders can any of them be examined at cluster level? – we intend to look into this in subsequent work

8) what is the effect of lumping the data together at EU level and is a sensitivity analysis at a subunit level informative – we intend to look into this in subsequent work

9) under 5s are at high risk of trachoma but are not usually treated with IVM. Is the availability of a reservoir of faecal material in all environments which has not come from an IVM treated individual not a concern for the hypothesis? – We are well aware of this, and intend to evaluate during subsequent work

I'd favour representing this information with a more determined analytical approach to confounding and less speculative conclusions. There is not really any evidence so far that this is anything to do with the reproductive capacity of M.sorbens, which, if anything, is known to be quite robust. The observation itself is interesting and potentially important- it would merit a more careful examination of all the potential explanations both analytically and in discussion
