# Supplementary material for: Potential mitigating role of ivermectin on the spread of Chlamydia trachomatis by Musca sorbens
Source: PLoS Negl Trop Dis. 2023 Oct 26;17(10):e0011662. doi: 10.1371/journal.pntd.0011662 (PMC10602231; doi:10.1371/journal.pntd.0011662)
Supplement: Attachment — Submitted filename: Response to reviewers round 2.docx [file pntd.0011662.s002.docx]

Dear reviewers

We thank you for the inputs you have made to the paper and the effort that you have made in improving this work.

We have worked through all the suggestions and agree that they improve the paper. Their acceptance is visible in the track changes included in this resubmission, so we shall not work through them individually here.

There are a few listed below that we have included some additional information or justification on.

We deeply appreciate the guidance and improvement that has come as a result of both rounds of review.

Best wishes

Richard

137-138: “A backward selection procedure was used to retain the candidate model with the best subset of variables.” The word “best” here is vague. Do the authors mean “with the subset of variables that demonstrated statistically significant associations with the outcome of interest, using an alpha of 0.05”?

We have now clarified the procedure in the manuscript. The criterion used in the backwards selection was the AIC, not the alpha.

139: I am not sure what “and numerical issues” means. Should these words be deleted? Or do the authors want to say that “a history of receiving ivermectin completely predicted an absence of active trachoma recrudescence”?

We meant the logistic regression model failed to converge, as expected, due to the quasi-complete separation in the dataset for recrudescence as all EUs that received ivermectin had no recrudescent trachoma, since the maximum likelihood estimate does not exist in this case. We have clarified in response to your comments.

228: Suggest delete “In a multivariable model with all variables entered simultaneously, the association between IVM and persistence status is no longer statistically significant (p=0.15).” and change the opening of the subsequent sentence to read “Using the backward selection process, the best candidate multivariable model…”. The final multivariable model is the one that readers are interested in, not all possible candidate models.

We prefer to remain cautious in our conclusions, but stress that they are highly encouraging.
The reason we used two approaches is as follows

1) We entered all the potential confounders for which data were available in the model with simultaneous entry to examine the magnitude and significance of the association between persistence and IVM adjusting for all potential confounders.
2) We supplemented this approach with an automated variable selection procedure to explore what variables a more parsimonious model would contain.

We found it encouraging that IVM was retained at this stage. However, our cautious approach is built on our variable selection process being purely data-driven and based on a statistical criterion as this might not adequately reflect the mechanisms at play in the relationships between the independent variables and the outcome. Hence further research being needed.

237: Similarly, suggest delete the material undermining the hypothesis that reads “The results are not clear-cut as association becomes non-significant.” If you are in any doubt about how to undertake and interpret the multivariable modelling, I strongly recommend enlisting additional advice from an experienced modeller!
Please see above.
